# Cost of off-label antibiotic therapy for bone and joint infections: a 6-year prospective monocentric observational cohort study in a referral centre for management of complex osteo-articular infections

**DOI:** 10.5194/jbji-6-337-2021

**Published:** 2021-09-07

**Authors:** Truong-Thanh Pham, Eugénie Mabrut, Philippe Cochard, Paul Chardon, Hassan Serrier, Florent Valour, Laure Huot, Michel Tod, Gilles Leboucher, Christian Chidiac, Tristan Ferry

**Affiliations:** 1 Infectious Diseases Department, Croix-Rousse Hospital, Hospices Civils de Lyon, 69004 Lyon, France; 2 French Referral Centre for complex Bone and Joint Infections, CRIOAc Lyon, 69000 Lyon, France; 3 Division of Infectious Diseases, Department of Medicine, Geneva University Hospitals, 1205 Geneva, Switzerland; 4 Hauteville Public Hospital Centre, 01110 Hauteville-Lompnes, France; 5 Val Rosay Rehabilitation Centre, 69370 Saint-Didier-Au-Mont-d'Or, France; 6 Pôle de Santé publique, Hospices Civils de Lyon, 69003 Lyon, France; 7 Cellule Innovation, Département de la Recherche Clinique et de l'innovation, Hospices Civils de Lyon, 69003 Lyon, France; 8 Centre International de Recherche en Infectiologie (CIRI), Inserm U1111, CNRS UMR5308, ENS de Lyon, UCBL1, 69007 Lyon, France; 9 Service de Pharmacie, Hospices Civils de Lyon, Lyon, France

## Abstract

**Introduction**: Costs related to bone and joint infection (BJI) management are increasing worldwide, particularly due to the growing use of off-label antibiotics that are expensive treatments (ETs), in conjunction with increasing incidence of multi-drug-resistant pathogens. The aim of this study was to evaluate the whole costs related to these treatments during the patient route, including those attributed to the rehabilitation centre (RC) stay in one regional referral centre in France. The total annual cost of ETs for managing complex BJIs in France was then estimated.

**Material and methods**: A prospective monocentric observational study was conducted from 2014 to 2019 in a referral centre for BJI management (CRIOAc – Centre de Référence des Infections OstéoArticulaires complexes). Costs related to expensive treatments (“old” ETs, i.e. ceftaroline, ertapenem, daptomycin, colistin, tigecycline, and linezolid and “new” ETs, defined as those used since 2017, including ceftobiprole, ceftazidime-avibactam, ceftolozane-tazobactam, tedizolid, and dalbavancin) were prospectively recorded. In all cases, the use of these ETs was validated during multidisciplinary meetings.

**Results**: Of the 3219 patients treated, 1682 (52.3 %) received at least one ET, and 21.5 % of patients who received ET were managed in RCs. The overall cost of ETs remained high but stable (EUR 1 033 610 in 2014; EUR 1 129 862 in 2019), despite the increase of patients treated by ETs (from 182 in 2014 to 512 in 2019) and in the cumulative days of treatment (9739 to 16 191 d).

Daptomycin was the most prescribed molecule (46.2 % of patients in 2014 and 56.8 % in 2019, with 53.8 % overall), but its cost has decreased since this molecule was genericized in 2018; the same trend was observed for linezolid. Thus, costs for old ETs decreased overall, from EUR 1 033 610 in 2014 to EUR 604 997 in 2019, but global costs remained stable due to new ET utilization accounting for 46.5 % of overall costs in 2019. Tedizolid, used as suppressive antimicrobial therapy, represented 77.5 % of total new ET costs. In our centre, dalbavancin was never used.

The cost paid by RCs for ETs and the duration of ET remained stable overall between 2016 and 2019.

**Conclusions**: A high consumption of off-label ET is required to treat patients with BJIs in a CRIOAc, and the consequence is a high cost of antimicrobial therapy for these patients, estimated to be almost EUR 10 million in France annually. Costs associated with ET utilization remained stable over the years. On the one hand, the introduction of the generic drugs of daptomycin and linezolid has significantly decreased the share of old ETs, but, on the other hand, the need for new ETs to treat infections associated with more resistant pathogens has not led to decrease in the overall costs. A drastic price reduction of generic drugs is essential to limit the costs associated with more complex BJIs.

## 1 Introduction

Bone and joint infections (BJIs) are constantly increasing worldwide (Premkumar et al., 2021; Kehrer et al., 2014; Kremers et al., 2015; Rutherford et al., 2016) and have a significant clinical and economic burden. In France, the incidence is estimated at 70 per 100 000 persons per year, and in 2013, the total direct cost of treating BJIs was estimated at EUR 421 million (USD 509 million), including EUR 11 960 (USD 14 460) per hospital stay (Lemaignen et al., 2021; Laurent et al., 2018; Ferry et al., 2019).

Infections involving an internal device account for up to 57.7 % of complex BJIs (Lemaignen et al., 2021). They are more costly than native BJIs. Indeed, at least one surgery is required, the prevalence of multi-drug-resistant bacteria is higher, and the use of expensive antibiotics is more frequent (Hackett et al., 2015).

Peri-prosthetic joint infections (PJIs) represent the vast majority of these implant infections, and more than one-third of all BJIs (Lemaignen et al., 2021). They are increasing, with a significant morbidity and mortality rate, and high costs. In the USA, projections for 2030 estimate that the annual number of PJIs (hip and knee) could rise to more than 66 000 cases per year, with a total cost of more than EUR 1.53 billion (USD 1.85 billion) (Premkumar et al., 2021).

The CRIOAc (Centre de Référence des Infections OstéoArticulaires complexes) network, composed of nine regional referral centres, was implemented in France in 2008 by the General Directorate for the Provision of Healthcare (Direction Générale de l'Offre de Soins; DGOS) for the management of complex BJIs. The complex nature is defined by a patient who presents one or more of the following criteria: (i) relapse, (ii) host-related criteria, e.g. anaesthetic risk terrain, allergy limiting therapeutic management, and history limiting and/or modifying surgical management, (iii) surgical-related criteria, e.g. the need for bone resection or complex bone and/or soft tissue reconstruction, and (iv) pathogen-related criteria, which may be multi-drug-resistant with limited therapeutic possibilities (Ministère De La Santé, 2010).

Antibiotic resistance has an impact on the cost of BJIs as it limits the use of orally adequate available drugs and may promote less active or more expensive drug administration. In the USA, the cost related to treatment of BJIs is significantly higher for methicillin-resistant *S. aureus* infections (approximately EUR 88 755 or USD 107 264) than for methicillin-susceptible *S. aureus* infections (approximately EUR 56 300 or USD 68 053; Parvizi et al., 2010). The growing incidence of resistant pathogens involved in BJIs, such as multi-drug-resistant (MDR) coagulase-negative staphylococci or MDR gram-negative pathogens (Titecat et al., 2013; Da Silva and Salles, 2021), and the occurrence of side effects under conventional treatment are leading to the increasing use of off-label molecules for the treatment of BJIs.

These antibiotics have sometimes been on the market for more than 10 years and have only been validated for skin and soft tissue infections (anti-gram-positive antibiotics), and urinary tract or intra-abdominal infections (anti-gram-negative antibiotics). The vast majority of them are expensive treatments (ETs). Due to their potential high cost, their use has to be validated during multidisciplinary meetings; therefore, they may be associated with a significant increase in costs of treating BJIs. This could also be a barrier to management in rehabilitation centres (RCs).

There are no precise data in France concerning the volume and cost of prescriptions for ETs. The main objective of this study was to estimate the whole cost of off-label ETs over time for patients treated at CRIOAc in Lyon. We included RCs and outpatient prescriptions in this descriptive analysis.

## 2 Method

### 2.1 Study design

A prospective monocentric cohort study was conducted at CRIOAc in Lyon (https://www.crioac-lyon.fr, last access: 14 June 2021) and included patients managed for an osteo-articular infection between 1 January 2014 and 31 December 2019. Those patients who refused to participate in the study were excluded. The clinical situation of every patient referred to this referral centre was discussed during multidisciplinary meetings, and the use of every off-label antibiotic was also validated at these meetings.

### 2.2 Study variables and definitions

Patient and BJI characteristics and prescribing patterns were collected during each visit, including the prescription of antibiotics by RCs or in an outpatient setting. Data were collected on patient's age, sex, body mass index (BMI), American Society of Anesthesiologists (ASA) score, presence or absence of implant, infection type (peri-prosthetic joint infection, osteosynthesis-associated infection, native osteomyelitis, and septic arthritis), time between hardware placement and symptom onset (<1 month – acute infection; 1–3 months – subacute infection; >3 months – chronic infection), whether the patient was managed by a RC or not, and the start and end dates of antibiotics and their dosage.

ETs were separated into two groups, i.e. “old” and “new”, according to their dates of use. Old ETs were ceftaroline, ertapenem, daptomycin, colistin, tigecycline, and linezolid. New ETs, defined as those used from 1 January 2017 onwards, included ceftobiprole, ceftazidime-avibactam, ceftolozane-tazobactam, tedizolid, and dalbavancin. Each ET was prescribed as empirical, targeted, or suppressive therapy.

Peri-prosthetic joint infections (PJIs) and osteosynthesis-associated infections were defined according to the MusculoSkeletal Infection Society (MSIS) 2018 criteria (Parvizi et al., 2018) and the fracture-related infections (FRI) consensus group, respectively (Metsemakers et al., 2018). Osteomyelitis was defined by the Infectious Diseases Society of America (IDSA) definition (Berbari et al., 2015) and septic arthritis by modified Newman's criteria (Mathews et al., 2010).

As RCs were not equipped with the same computerized prescription program, they were asked to fill out a table with the dates of admission and exit of their institution. A cross-reference was made between ET dispensation dates and patient care in RCs, making it possible to calculate the exact patient length of stay and duration of treatment (DOT) in RCs.

Eventually, as we know the number of inhabitants that are referred to our centre, the total annual cost attributed to ETs in France was estimated, using the 2018 census for calculation.

### 2.3 Statistical analysis

Categorical variables were described by counts and percentages, while mean and standard deviation or median and interquartile range (IQR) were used to summarize continuous variables. Charts were made with Microsoft Excel for Apple Mac (version 16.48). Description analyses were done with Stata 16.1 (StataCorp LLC, Texas 77845, USA).

## 3 Results

### 3.1 Patient characteristics

Between 1 January 2014 and 31 December 2019, 3219 patients were managed at our referral centre with the following distribution: 410 patients in 2014, 473 in 2015, 524 in 2016, 564 in 2017, 581 in 2018, and 652 in 2019. Of these, 1705 received one or more ET. A total of 23 were excluded from the study (eight declined to participate; 15 did not receive information). Thus, 1682 (52.5 %) were included in the study analysis, i.e. 182 patients (44.4 %) in 2014, 214 (45.2 %) in 2015, 220 (42.0 %) in 2016, 249 (44.1 %) in 2017, 305 (52.5 %) in 2018, and 512 (78.5 %) in 2019.

Patients who received ETs were predominantly male (n=1048; 62.3 %), with a median age of 64.0 years ( 50.0–76.0 IQR) and a median BMI of 25.8 kg/m${}^{2}$ (22.8–30.3 IQR). The majority of BJIs were with an internal device (n=975; 58.0 %) with a predominance of PJIs (n=581; 60.6 % of implant-associated BJIs) and with an equal proportion of acute (n=434; 25.8 %) and chronic (n=430; 25.6 %) infections. The lower limbs were most frequently affected (n=1103; 64.5 %; see Table 1).

**Table 1:** Patient and infection characteristics. IQR – interquartile range; BMI – body mass index; ASA – American Society of Anesthesiologists; PJI – peri-prosthetic joint infection; OAI – osteosynthesis-associated infection; ORL – otorhinolaryngology.

Patient characteristics	n=1682
Sex (male), n (%)	1048 (62.3)
Age (years), median (IQR; range)	64.0 (50.0–76.0; 17.0–91.0)
BMI (kg/m${}^{2}$), median (IQR; range)	25.8 (22.8–30.3; 16.2–49.1)
ASA score, median (IQR)	2 (2–3)
Infection characteristics	n=1682
Presence of internal device, n (%)	975 (58.0)
PJI, n (%)	568 (33.2)
OAI, n (%)	381 (22.7)
PJI + OAI, n (%)	23 (1.4)
Other type of device, n (%)	13 (0.8)
Native (without device), n (%)	707 (42.0)
Infection location	n=1709
Lower limbs, n (%)	1,103 (64.5)
Upper limbs, n (%)	85 (5.0)
Pelvis, n (%)	183 (10.7)
Spine, n (%)	121 (7.1)
ORL, n (%)	114 (6.7)
Chest, n (%)	54 (3.2)
Skull, n (%)	37 (2.2)
Other location, n (%)	12 (0.7)
Infection chronology	n=1682
Acute (<1 month), n (%)	434 (25.8)
Sub-acute (≥1 month and ≤3 months), n (%)	111 (6.6)
Chronic (>3 months), n (%)	430 (25.6)
Not applicable (without device)	707 (42.0)
Microbiology	n=3975
Gram-positive pathogens, n (%)	2534 (63.7)
Gram-negative pathogens, n (%)	1067 (26.8)
*Pseudomonas aeruginosa*	200 (5.0)
Sterile, n (%)	374 (9.4)

The part of gram-positive pathogens increased during the study period (54.8 % to 65.6 %); it contrasted with decrease in gram-negative BJIs (33.2 % to 24.4 %); over the 6 years, gram-positive bacteria represented more than double the gram-negative bacteria (n=2534 (63.7 %) and n=1067 (26.8 %), respectively). *Pseudomonas aeruginosa* BJIs remained stable over the 6-year study period (approximately 5 %; see Table 2).

**Table 2:** Type of pathogens. BJIs – bone and joint infections.

	2014	2015	2016	2017	2018	2019	Total
Gram-positive pathogens, n (%)	241 (54.8)	298 (60.6)	324 (63.2)	381 (65.9)	477 (67.0)	813 (65.6)	2534 (63.7)
Gram-negative pathogens, n (%)	146 (33.2)	158 (32.1)	154 (30.0)	148 (25.6)	158 (22.2)	303 (24.4)	1067 (26.8)
*Pseudomonas aeruginosa*	20 (4.5)	22 (4.5)	25 (4.9)	39 (6.7)	36 (5.1)	58 (4.7)	200 (5.0)
Culture-negative BJIs, n (%)	53 (12)	36 (7.3)	35 (6.8)	49 (8.5)	77 (10.8)	124 (10.0)	374 (9.4)
Total, n	440	492	513	578	712	1240	3975

### 3.2 Duration and types of antimicrobial therapy

**Figure 1:**
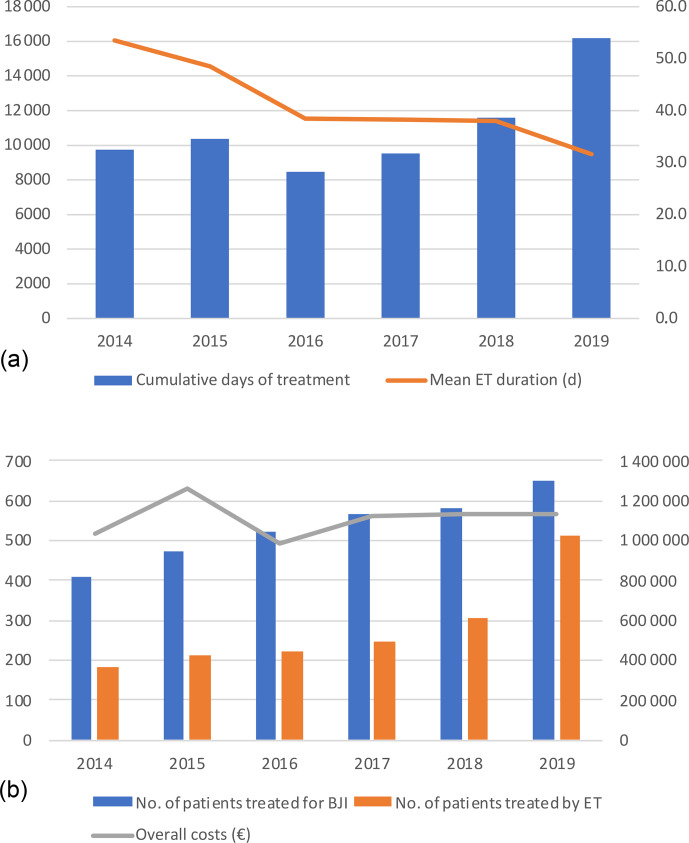
**(a)** Cumulative days of expensive treatments and mean duration. **(b)** Number of patients treated (blue) in the referral centre with expensive treatment (orange) and global costs (line). ET – expensive treatment; BJI – bone and joint infection.

Days of treatment with ETs remained stable between 2014 and 2018 but increased by almost 1.5 times in 2019 to reach 16 191 d, which is also in line with the increase in patients treated in the referral centre (n=652 vs. 410) and the increasing proportion of patients requiring ET (n=512 (78.5 %) vs. 182 (44.4 %)). The average DOT was reduced by 21.9 d during the study, i.e. 53.5 in 2014 to 31.6 d in 2019. (Table 3; Fig. 1).

**Table 3:** Number of patients treated in our referral centre and the duration and costs related to expensive treatment. BJIs – bone and joint infections; ET – expensive treatment; RC – rehabilitation centre.

	2014	2015	2016	2017	2018	2019	Total
No. of patients treated for BJIs	410	473	524	564	581	652	3204
No. of patients treated with ET, n (%)	182 (44.4)	214 (45.2)	220 (42.0)	249 (44.1)	305 (52.5)	512 (78.5)	1682 (52.5)
Days of ET	9739	10 377	8462	9525	11 580	16 191	65 874
Mean duration of ET per patient (d)	53.5	48.5	38.5	38.3	38.0	31.6	39.2
Overall costs (EUR)	1 033 610	1 265 520	984 764	1 120 338	1 132 724	1 129 862	6 666 818
Old ET total costs (EUR)	1 033 610	1 265 520	984 764	1 005 378	921 843	604 997	5 816 112
New ET total costs (EUR)	0	0	0	114 960	210 881	524 865	850 706
Percent of new ET costs/overall costs (%)	0	0	0	10.3	18.6	46.5	12.8
RC							
No. of patients treated in RC, n (%)	50 (27.5)	49 (22.9)	22 (10.0)	63 (25.3)	91 (29.8)	87 (17.0)	362 (21.5)
Days of ET in RC	2092 (21.5)	1530 (14.7)	1473 (17.4)	1452 (15.2)	1983 (17.1)	2406 (14.9)	10 936 (16.6)
Mean duration of ET per patient in RC (d)	41.8	31.2	67.0	23.0	21.8	27.7	30.2
Costs of ET in RC (EUR)	218 836	173 891	191 098	192 229	200 651	113 848	1 090 553
Percent of ET in RC costs/overall costs (%)	21.2	13.7	19.4	17.2	17.7	10.1	16.4

Daptomycin was the most commonly prescribed drug, accounting for almost half of treatment days (n=32503 d; 49.3 %), followed by ertapenem (n=11914 d; 18.1 %), and linezolid (n=10409 d; 15.8 %). Use of ertapenem and colistin decreased over the 6-year period at the time of the introduction of ceftazidime-avibactam and ceftolozane-tazobactam; utilization of these drugs remained marginal. The remaining ETs were also modestly used, but it is worth mentioning that tedizolid accounted for 14.5 % of ETs in 2019, mainly due to its use as a suppressive antimicrobial therapy (SAT). Indeed, unlike other centres, dalbavancin was not chosen as potential treatment for BJIs and was particularly not chosen as SAT (Table 4).

**Table 4:** Expensive treatments, with the duration and costs by molecule and by year. NA – not applicable; DAP – daptomycin; ETP – ertapenem; CST – colistin; TGC – tigecycline; LZD – linezolid; CPT – ceftaroline; TZD – tedizolid; C/T – ceftolozane-tazobactam; CZA – ceftazidime-avibactam; BPR – ceftobiprole.

	DAP	ETP	CST	TGC	LZD	CPT	TZD	C/T	CZA	BPR	Total
2014											
No. of patients	84	37	12	11	37	1	NA	NA	NA	NA	182
(%)	(46.2)	(20.3)	(6.6)	(6.0)	(20.3)	(0.5)	NA	NA	NA	NA	
No. of days	4355	2981	422	704	1236	41	NA	NA	NA	NA	9739
(%)	(44.7)	(30.6)	(4.3)	(7.2)	(12.7)	(0.4)	NA	NA	NA	NA	
Costs (EUR)	609 340	167 051	28 404	70 400	150 975	7440	NA	NA	NA	NA	1 033 610
(%)	(59.0)	(16.2)	(2.7)	(6.8)	(14.6)	(0.7)	NA	NA	NA	NA	
2015											
No. of patients	106	37	11	9	48	3	NA	NA	NA	NA	214
(%)	(49.5)	(17.3)	(5.1)	(4.2)	(22.4)	(1.4)	NA	NA	NA	NA	
No. of days	5267	2046	756	418	1551	339	NA	NA	NA	NA	10 377
(%)	(50.8)	(19.7)	(7.3)	(4.0)	(14.9)	(3.3)	NA	NA	NA	NA	
Costs (EUR)	825 198	115 255	46 008	41 300	183 540	54 219	NA	NA	NA	NA	1 265 520
(%)	(65.2)	(9.1)	(3.6)	(3.3)	(14.5)	(4.3)	NA	NA	NA	NA	
2016											
No. of patients	129	26	14	13	34	4	NA	NA	NA	NA	220
(%)	(58.6)	(11.8)	(6.4)	(5.9)	(15.5)	(1.8)	NA	NA	NA	NA	
No. of days	4748	1018	769	714	1170	43	NA	NA	NA	NA	8462
(%)	(56.1)	(12.0)	(9.1)	(8.4)	(13.8)	(0.5)	NA	NA	NA	NA	
Costs (EUR)	737 774	64 251	49 029	85 200	42 651	5859	NA	NA	NA	NA	984 764
(%)	(74.9)	(6.5)	(5.0)	(8.7)	(4.3)	(0.6)	NA	NA	NA	NA	
2017											
No. of patients	129	28	16	16	45	3	1	8	2	1	249
(%)	(51.8)	(11.2)	(6.4)	(6.4)	(18.1)	(1.2)	(0.4)	(3.2)	(0.8)	(0.4)	
No. of days	4495	1885	441	627	1538	120	3	375	20	21	9525
(%)	(47.2)	(19.8)	(4.6)	(6.6)	(16.1)	(1.3)	(0.0)	(3.9)	(0.2)	(0.2)	
Costs (EUR)	744 241	96 214	41 575	94 050	6978	22 320	609	102 765	10 200	1386	1 118 952
(%)	(66.5)	(8.6)	(3.7)	(8.4)	(0.6)	(2.0)	(0.1)	(9.2)	(0.9)	(0.1)	
2018											
No. of patients	166	31	8	15	70	3	5	3	3	1	305
(%)	(54.4)	(10.2)	(2.6)	(4.9)	(23.0)	(1.0)	(1.6)	(1.0)	(1.0)	(0.3)	
No. of days	5627	2167	220	517	2010	136	734	125	27	17	9525
(%)	(48.6)	(18.7)	(1.9)	(4.5)	(17.4)	(1.2)	(6.3)	(1.1)	(0.2)	(0.1)	
Costs (EUR)	681 878	115 847	12 964	77 550	8308	25 296	124 046	69 750	13 770	3315	1 129 409
(%)	(60.4)	(10.3)	(1.1)	(6.9)	(0.7)	(2.2)	(11.0)	(6.2)	(1.2)	(0.3)	
2019											
No. of patients	291	29	10	7	137	2	24	4	6	2	512
(%)	(56.8)	(5.7)	(2.0)	(1.4)	(26.8)	(0.4)	(4.7)	(0.8)	(1.2)	(0.4)	
No. of days	8011	1817	497	343	2904	5	2347	110	145	12	16 191
(%)	(49.5)	(11.2)	(3.1)	(2.1)	(17.9)	(0.0)	(14.5)	(0.7)	(0.9)	(0.1)	
Costs (EUR)	442 369	86 989	20 976	35 920	17 818	925	406 683	41 990	73 863	2329	1 127 533
(%)	(39.2)	(7.7)	(1.9)	(3.2)	(1.6)	(0.1)	(36.1)	(3.7)	(6.6)	(0.2)	
Total											
No. of patients	905	188	71	71	371	16	30	15	11	4	1682
(%)	(53.8)	(11.2)	(4.2)	(4.2)	(22.1)	(1.0)	(1.8)	(0.9)	(0.7)	(0.2)	
No. of days	32 503	11 914	3105	3323	10 409	684	3084	610	192	50	65 874
(%)	(49.3)	(18.1)	(4.7)	(5.0)	(15.8)	(1.0)	(4.7)	(0.9)	(0.3)	(0.1)	
Costs (EUR)	4 040 800	645 607	198 956	404 420	410 270	116 059	531 338	214 505	97 833	7030	6 659 788
(%)	(60.7)	(9.7)	(8.0)	(6.1)	(6.2)	(1.7)	(3.0)	(3.2)	(1.5)	(0.1)	

### 3.3 Expensive treatment costs

**Figure 2:**
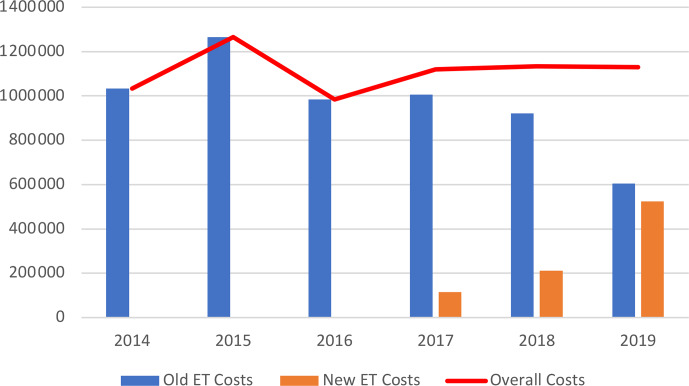
Costs (EUR) related to old and new expensive treatments and total costs. ET – expensive treatment.

The overall costs related to ETs remained stable between 2014 and 2019 (EUR 1 033 610 and EUR 1 129 862, respectively), while the number of patients treated, and those for whom ETs were needed, increased. Without new ETs, the costs associated with old ETs would have almost halved between 2014 and 2019 (EUR 1 023 890 to EUR 604 997), with this being related to the appearance of generic molecules (Tables 3 and 4; Fig. 2).

**Figure 3:**
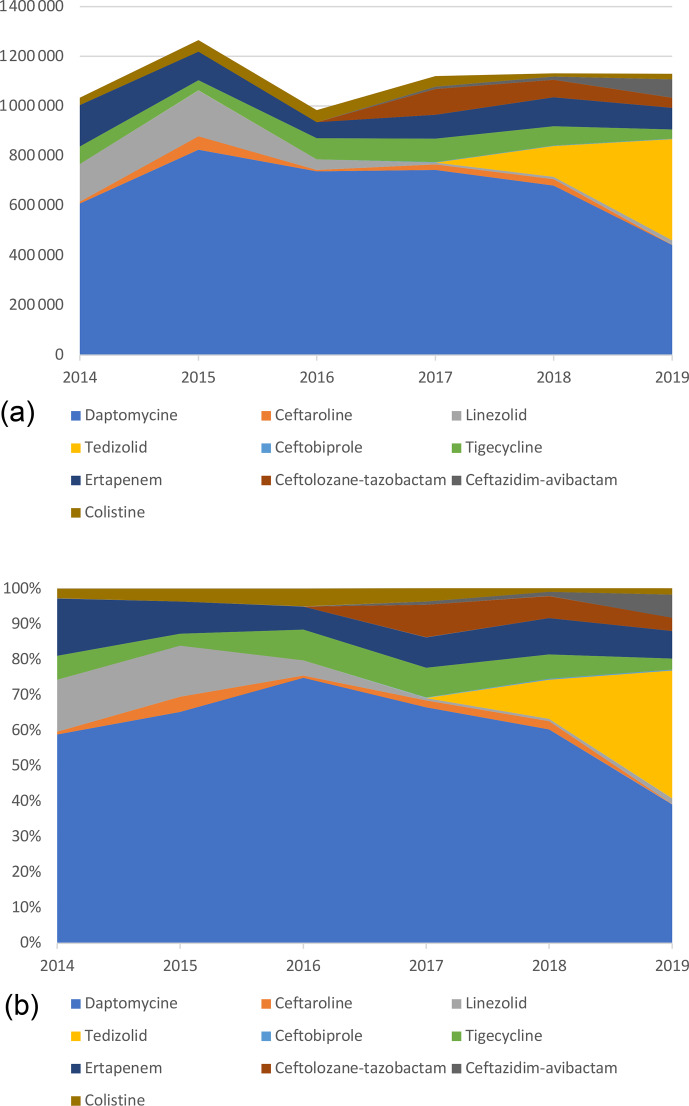
Cumulative **(a)** and proportional **(b)** costs (EUR) related to expensive treatments, by molecule.

Daptomycin represented the majority of ET costs (60.6 % of total costs) but was decreasing since 2016 with the introduction of generic molecules. Indeed, by genericizing the molecule, the price went down from around EUR 150 to EUR 55. In 2019, it corresponded only to 39.2 % of the total costs, whereas in 2016 it was 74.9 %, while DOT had doubled (Table 4; Fig. 3).

The same trend was observed for linezolid with a significant decrease in price, from EUR 122 for 37 patients in 2014 (1236 d) to around EUR 5 in 2019 for 137 patients (2904 d). Despite the 2-fold increase in the number of days, total costs related to linezolid decreased by more than 10 times (EUR 150 975 to EUR 17 818).

Ertapenem costs decreased, which is related to the use of new ETs. Indeed, ceftazidime-avibactam and ceftolozane-tazobactam have been prescribed since 2017. This use, however, remained marginal compared to treatments targeting gram-positive pathogens.

Eventually, costs related to tedizolid increased drastically in 2019, representing 36.0 % of the overall costs. This expensive molecule (EUR 175) has been frequently used as a SAT, resulting in long treatment duration and high costs. In 2019, it accounted for the vast majority (77.5 %) of new ET costs and treatment days (89.8 %).

### 3.4 Rehabilitation centre

Between 2014 and 2019, patients under ETs in RCs increased from 50 in 2014 to 87 in 2019. Nevertheless, the proportion of RC patients receiving ETs compared to the number of patients under ETs remained stable at 21.5 % (Table 3).

The length of stay in RCs decreased between 2014 and 2017 and then increased from 2092 d in 2014 to 2406 d in 2019.

The RC global costs related to ETs decreased over this period as follows: EUR 218 836 (21.2 % of global ET costs) in 2014, EUR 173 391 (13.7 %) in 2015, EUR 191 098 (19.4 %) in 2016, EUR 192 229 (17.2 %) in 2017, EUR 200 651 (17.7 %) in 2018, and EUR 113 848 (10.1 %) in 2019.

### 3.5 Estimation of the global cost in France

Our referral centre is the dedicated centre for the management of complex BJIs in the Auvergne-Rhône-Alpes region located in the east of France with 7 994 459 inhabitants. The other part of the country is covered by eight other CRIOAcs. With a total population in France of 68 014 000 inhabitants, the annual cost due to off-label antibiotics for the treatment of BJIs within the CRIOAc network in France would be almost EUR 10 million euros (EUR 9 612 462).

## 4 Discussion

Our study quantified the overall cost of off-label ETs used in BJIs over 6 consecutive years within a CRIOAc. The costs remained globally stable over the study period despite (1) an increasing number of managed patients, (2) a larger portion of these patients requiring ETs, and (3) an increase in cumulative treatment days. This is especially due to the introduction of generic molecules such as daptomycin and linezolid, whose prices, unlike ertapenem, have decreased considerably.

This decrease in costs related to these generic drugs is, however, counterbalanced by the appearance of multi-drug-resistant bacteria requiring the use of more expensive, new ETs such as tedizolid. This oxazolidinone antibiotic is similar to linezolid in terms of the spectrum of activity, and it remains active against gram-positive multi-resistant pathogens (Carvalhaes et al., 2019). Unlike linezolid, this antibiotic appears to be better tolerated, with less myelotoxicity and neuropathy and fewer drug–drug interactions (Ferry et al., 2018; Douros et al., 2015). This makes it an excellent choice for SAT, especially for patients with MDR gram-positive PJIs for whom no oral options were available before the market launch of this drug (Ferry et al., 2021). Yet, it remains expensive; in 2019, it accounted for one-third of ET costs and for less than 15 % of cumulative treatment days. It should be noticed that this antibiotic is not available in all countries, such as Switzerland, and that some centres use dalbavancin as a SAT; in our centre, however, it was decided to use tedizolid because it can be taken by mouth, unlike dalbavancin, which requires an injection to be administered in the hospital every 1 or 2 weeks in France (Dinh et al., 2019).

Daptomycin was the most frequently used antibiotic in our study, accounting for almost half of the cumulative days and almost 60 % of the total costs. However, its share of costs has drastically decreased since the introduction of generic drugs in 2018, with a high drop in its price in 2019. It is worth noticing that this ET has been used more since 2018 when the combination of piperacillin-tazobactam and vancomycin was shown to increase the risk of acute renal failure (Triffault-Fillit et al., 2018, 2020).

Generic drugs are defined as bio-equivalent replicas of brand name drugs, containing the same active molecules, with identical quality, safety, and efficacy profiles. Only inactive ingredients, like colouring, flavouring, and stabilizing agents can differ (Wouters et al., 2017). Generic drugs can be approved for sale when relevant patents and legal exclusivities have expired (generally 20 years) or when the patent owner waives their rights (Gulsen Oner and Polli, 2018). Yet, generic drugs have been associated with notable monetary savings. For instance, about 90 % of all prescriptions were filled using a generic drug product in 2019 in the USA, and USD 313 billion could be saved (Association for Accessible Medicines, 2021). Unfortunately, these results do not represent what is has been achieved in other countries. In Switzerland, the proportion of prescriptions filled with generic drugs is only about 17 % to 23 %, while in France it is about 30 % to 40 % – far from Germany and Great Britain, with 80 % to 82 % and 83 % to 85 %, respectively (Wouters et al., 2017; Decollogny et al., 2011; Organisation for Economic Co-Operation and Development (OECD), 2019). Nevertheless, without the generic molecules of daptomycin and linezolid, the costs associated with ETs during the study period could not have been stable and would have increased dramatically, especially in 2019 with the use of tedizolid, ceftolozane-tazobactam, and ceftazidime-avibactam.

With regard to RCs, their part of the cost was not the majority. The proportion of patients under ETs, the cost paid by RCs for ETs, and the number of cumulative days remained globally stable over the study period.

This study has some other limitations. Indeed, the choice of which antibiotic was an ET was arbitrary and could, therefore, change from one country to another. Moreover, it was a monocentric analysis; the prescription of any antibiotic is, therefore, linked to the practices of the centre and is, therefore, not totally generalizable. Nevertheless, as the patients were treated in a referral centre, it can be assumed that these practices are not so far from the other CRIOAcs in France. Moreover, CRIOAcs are located in hospitals that are mostly members of the same central purchasing group; therefore, they benefit from the same prices negotiated by the laboratories marketing the antibiotics.

Finally, the costs related to antibiotics reflect only part of the costs generated by BJIs. However, in France, the costs are covered by the insurance companies on a fixed-price basis according to the diagnosis code of the disease. Therefore, excess costs related to ETs are supported by the hospitals and also by the healthcare system.

In conclusion, off-label ET use is common for treating BJIs in a referral centre in France, with a huge total cost (estimated to EUR 10 million per year in France), given the entire course of the patient from hospital to RCs and/or outpatient settings. With the introduction of generic molecules of daptomycin and linezolid, overall costs remained stable over the years in our study. Thus, with the rising number of multi-drug-resistant infections, the production of generic antibiotics at low cost is essential to limit the financial burden of the management of BJIs.

## Data Availability

The data that support the findings of this study are available from the corresponding author upon reasonable request.
